# Specialism and Individualism

**Published:** 1892-08-13

**Authors:** 


### The Doctor's Armchair.

### SPECIALISM AND INDIVIDUALISM.

This is the day of specialists. Kings and their favourite ministers had a long innings as directors-general of human affairs. In the old world prophets also often essayed the task of directorship; and in the modern, priests have striven hard to mount the hobby-horse of universal authority, and to guide him whithersover they would. The revolution of the wheel of development has now placed the specialist uppermost, and he takes as kindly to his new position and honours as does a male infant from the nursery to his first ride on the back of a seaside donkey.

The specialist is destined to play, and indeed is already playing, a considerable part in our every-day business. No schoolgirl can shed her milk-teeth without him; nor can even her marriageable elder sister keep her nails beautiful unless she can secure his aid. The specialist determines for us the constitution of the sun; and he also, for a consideration, cuts our corns. Such, and so wide, is the range of his activity! Before Carlyle, it was the custom to worship and admire almost everything that had the boldness to claim worship and admiration. Carlyle, too, could worship, but he insisted upon having a good, long, critical look even at his heroes. Things professing to be heroic, which he found upon examination to be unheroic, he cast down and trampled under his feet. It is an excellent rule which insists upon an adequate examination of a hero. It saves the world from too often making a fool of itself; and it keeps common-place persons on the safe level of the common ground, instead of lifting them to a giddy pinnacle, on which they cannot stand, and from which they must inevitably tumble to a deeper depth of insignificance than that which they occupied before.

In such a critical, dispassionate, but not unkindly spirit, let the modern specialist be examined. In the first place, it is clearly obvious that the specialist is a man—unless, indeed, he happens to be a woman—and even then he may, on the authority of the Laureate, be described as a "lesser man." He is a man—not a demigod. He wears a coat and trousers, and generally also a hat, though it may on occasion be a bad one. Not only is he a man, but he was once a boy, or a girl. There was a time when, like other common mortals, he was not a specialist; he knew nothing even of his own speciality, his astronomy or his chiropody. It is a distinct encouragement to the ordinary person to know that the clay-bed from which he himself was fashioned was the source and origin of the specialist; and that if he, the common man, had happened to be put in youth into one of the manifold moulds which give concrete form to the various specialisms, he too might, could, and would have been an astronomer, a biologist, or a maker of artificial teeth.

Besides, let it not be forgotten that there is a true and real sense in which every industrious human being is a specialist. The ploughman follows a specialism of the most fundamental kind. Compared with him the tooth-maker is insignificant, and the astronomer a mere chimney-piece ornament. There are hundreds of old specialisms, with their millions of specialists, which never think of setting up a claim to peculiar authority or honour. It is your new specialism, and your specialist without an ancestry, which make such a loud noise in the world. This cannot last. Does anybody suppose that a chemist will ever again send an electrical shock through the whole world as Professor Tyndal did from the presidential chair of the British Association? When Professor Huxley now reiterates his undying sympathy with the owners of Galilean pigs, and his sense of the enormity of the conduct of certain devils who persuaded some of those animals to plunge down a steep place into the sea, he can no longer extort a mild protest from Dr. Wace, nor even bring into action the sting of the Duke of Argyll. There was a time when Huxley's theological rattle aroused a startled and terrified world. The world now knows it to be a rattle—" that, and nothing more."

The common man will have to assert his rights once more. It was the common man who gave Shakespeare his permanent place in literature, and it was the common man also who insisted that *Paradise Lost* was one of the world's greatest poems, although a publisher's reader had valued it at ten pounds. Specialists desire to divide the world of men and women into flocks of sheep, of which they themselves shall be the honoured, enriched, and crook-bearing shepherds. When a specialist speaks let no dog bark. That is the note of the times. It is impossible, *messieurs les specialistes!* We value your services very much, but they are too dear at the price you ask for them. You are generally good craftsmen! Be content to be that, and do not pose as universal oracles on the strength of a few laboratory experiments. Besides—and this is a very shocking thing—it is positively unscientific to do so. What would Professor Huxley say if Dr. Wace were to correct his science on the question of the nervous system of the lobster? The whole world would be roused from slumber by the loudness of the great Professor's scornful laugh. But *ne sutor ultra crepidam*: Why should the apostle of the crayfish be anxious to figure as a father of the Christian Church? Moreover, he cannot do it. The huge common world has found him out. They think he is very good for the biological dishing up of crayfishes, but by no means the man for poetry, parable, and the deeper mysteries of the human mind. Voltaire was a much more brilliant genius than Huxley, but the world is doubtful whether his little candle of intellectual light is now worth even so much as snuffing. And, indeed, one cannot wonder at it. The stone effigy of the old man of Ferney looks by no means amiable as he sits on his insignificant pedestal in a semi-front garden at the back of the Sorbonne. He probably never made a single human being either happier or better in his life. The most insignificant general practitioner of physic who ever administered a well-timed blue pill has left a worthier record than that.

The world cannot afford to be overborne by its specialists. They must, as before, continue to stand at the bar of its critical judgment, and not be allowed to sit with the reins in their hands on the box seat of its car of progress. Specialists are like fire and water—good servants, but bad masters, and for the same reason, because their powers are usually bounded.

by their own specialisms. The commonest and most unlettered man is to be urged to think for himself on all questions which affect himself, and to trust his own judgment rather than to repose a blind confidence in any other person, be his name Voltaire, or Huxley, or Pope, or Smith. Just as the task of making a railway requires every individual navvy to remove his own proper number of shovelfuls of earth, so the due thinking out of the many and great problems which are set for the world's solution requires the efforts of every man's brains whom Nature has compelled to work out a man's destiny. Specialism! Yes! But specialism in its own place; and specialists as the servants of the hive. The bee community must, however, still be its own master, and guide its flight as it will.

